# Seaweed *Sargassum aquifolium* extract ameliorates cardiotoxicity induced by doxorubicin in rats

**DOI:** 10.1007/s11356-023-26259-z

**Published:** 2023-03-28

**Authors:** Rania Samir, Ekrami A. Hassan, Abdullah A. Saber, David S. A. Haneen, Eman M. Saleh

**Affiliations:** 1 Biochemistry Department, Faculty of Science, Ain Shams University, Abbassia, Cairo, 11566 Egypt; 2 Botany Department, Faculty of Science, Ain Shams University, Abbassia Square, Cairo, 11566 Egypt; 3 Chemistry Department, Faculty of Science, Ain Shams University, Abbassia, Cairo, 11566 Egypt

**Keywords:** Sodium alginate, Brown seaweeds, Doxorubicin, Cardiotoxicity, Oxidative stress, MAPKs, Apoptosis, Natural antioxidants

## Abstract

Doxorubicin (DOX) is a potent anticancer drug with adverse cardiotoxic effects. Alginates are multifunctional biopolymers and polyelectrolytes derived from the cell walls of brown seaweeds. They are nontoxic, biocompatible, and biodegradable, and hence, utilized in several biomedical and pharmaceutical applications. Here, we investigated the potential cardioprotective effect of thermally treated sodium alginate (TTSA), which was extracted and purified from the seaweed *Sargassum aquifolium*, in treating acute DOX cardiotoxicity and apoptotic pathways in rats. UV–visible spectroscopy, Fourier-transform infrared, and nuclear magnetic resonance (^1^H–NMR) spectroscopy techniques were used to characterize TTSA. CK-MB and AST levels in sera samples were determined. The expression levels of Erk-2 (MAPK-1) and iNOS genes were investigated by quantitative real-time polymerase chain reaction (qRT-PCR). The protein expression levels of Erk-2, anti-apoptotic p53, and caspase-3 were analyzed using western blotting and ELISA. For the in vivo studies, sixty rats were randomly divided equally into six groups and treated with DOX, followed by TTSA. We revealed that treatment with TTSA, which has low molecular weight and enhanced antioxidant properties, improved DOX-mediated cardiac dysfunction and alleviated DOX-induced myocardial apoptosis. Furthermore, TTSA exhibited a cardioprotective effect against DOX-induced cardiac toxicity, indicated by the increased expression of MAPK-1 (Erk2) and iNOS genes, which are implicated in the adaptive responses regulating DOX-induced myocardial damage. Moreover, TTSA significantly (*p* < 0.05) suppressed caspase-3 and upregulated anti-apoptotic protein p53 expression. TTSA also rebalanced the cardiomyocyte redox potential by significantly (*p* < 0.05) increasing the levels of endogenous antioxidant enzymes, including catalase and superoxide dismutase. Our findings suggest that TTSA, particularly at a dose of 400 mg/kg b.w., is a potential prophylactic supplement for treating acute DOX-linked cardiotoxicity.

**Supplementary Information:**

The online version contains supplementary material available at 10.1007/s11356-023-26259-z.

## Introduction

Doxorubicin (DOX) is a chemotherapeutic drug commonly used to treat solid and hematological malignancies. It is widely used to treat cancers, such as the stomach, breast, lung, ovaries, bladders, thyroids, soft tissue sarcoma, multiple myeloma, and Hodgkin’s lymphoma (Carvalho et al. 2009; Saleh et al. 2022). DOX inhibits macromolecular biosynthesis by intercalating with DNA. DOX stabilizes the topoisomerase II complex after breaking the DNA chain for replication, preventing the DNA double helix resealing and consequently terminating replication. DOX can also potentially produce free radicals, inducing DNA and cell membrane damage (Rivankar and Serums 2014).

DOX-based chemotherapy causes cardiovascular complications. The incidence of heart failure with a cumulative dose of DOX (400 mg/m^2^) has been reported to be approximately 3–5%, and can reach up to 48% when the total drug dose is increased to 700 mg/m^2^ (Zamorano et al. 2016). Oxidative stress generation is the main DOX-linked cardiotoxicity mechanism, linked to excessive production of free radicals and low levels of antioxidants (Saleh et al. 2022). DOX-induced oxidative stress might also activate apoptotic signaling pathways, resulting in cardiomyocyte apoptosis (Nitobe et al. 2003; Li et al. 2022). Moreover, DOX can stimulate nitric oxide (NO) production, causing dilated cardiomyopathy and congestive heart failure (Nakahara et al. 2018). DOX induces cancer cell senescence by phosphorylating p53, which upregulates p21, leading to cell cycle arrest (El-Far et al. 2021).

Seaweeds, also known as marine macroalgae, have been recently used in several industrial and pharmaceutical applications, such as novel food supplements (e.g., Alam et al. 2016; Al Adham et al. 2017; Leandro et al. 2020; Ibrahim et al. 2021, 2022) as they contain abundant multifunctional bioactive compounds such as polysaccharides, carotenoids, polyphenolics, antioxidants, dietary fibers, polyunsaturated fatty acids, vitamins, and minerals (Devi et al. 2011; Pereira 2020; Pradhan et al. 2021; Semaida et al. 2022). Several structurally diverse polysaccharides, such as alginates and fucoidan, found within the cell walls of brown seaweeds, are exploited for their powerful pharmaceutical and biomedical properties (Rashad and El-Chaghaby 2020; Rashedy et al. 2021; Saeed et al 2021; Ismail et al. 2022). The genus *Sargassum* C. Agardh and its related species, known as golden tides, are excellent sources of health-promoting antioxidants and alginate polysaccharides. Besides, their excellent nutritional properties make them ideal for pharmaceutical applications (Liu et al. 2012; Leandro et al. 2020; Seo et al. 2022). Alginate has mainly been utilized in pharmaceutical and food applications as nutraceuticals, microencapsulating, thickening, and stabilizing agents (Trica et al. 2019; Leandro et al. 2020). The edible macroalga *S. aquifolium* (Turner) C. Agardh is widespread in subtropical and tropical Indo-Pacific regions (Mattio et al. 2009; Guiry and Guiry 2022). This seaweed is among the predominant macroalgae found in the coastal waters of the Red Sea in Egypt (El Shafay et al. 2016; Talaat et al. 2022).

Chemically, alginates are linear co-polymers with homopolymeric blocks of 1,4-linked β-d-mannuronic acid (M) and α-l-guluronic acid (G) residues. Both monomers are arranged either in blocks of homopolymeric (M) or (G) and/or heteropolymeric (MG) or (GM) (Łabowska et al. 2019). The M segments have a flexible and linear structure, while the G segments have folded and stiff structural conformations (Cable 2009). The physical quality of alginates is determined by the percentage of the MM, GG, and MG blocks (Łabowska et al. 2019). Alginates with high G blocks have better gelling characteristics, while those with high M blocks have higher viscosity. In general, alginates with a high M/G ratio produce elastic gels, while alginates with a low M/G ratio form brittle gels (Fenoradosoa et al. 2010).

Natural alginates are derived from the cell walls of brown seaweeds (Sachan et al. 2009; El-Sheekh et al. 2022). Among the different types of alginates, sodium alginate is the most utilized polysaccharide for pharmaceutical studies (Szekalska et al. 2016). Alginates are used in pharmaceutical industries due to their biocompatibility, biodegradability, immunogenicity, and non-toxicity (Rinaudo 2014; Leandro et al. 2020). Given their potent anticancer and prebiotic effects, these valuable biopolymers are also used as food supplements (El-Sheekh et al. 2022). Although the extraction and purification of sodium alginate are straightforward, it is a multistage process (Lee and Mooney 2012).

This study aimed to assess the potential cardioprotective effects of thermally treated sodium alginate (TTSA) extracted from the brown seaweed *Sargassum aquifolium* against acute DOX cardiotoxicity using a rat model. We investigated the possible physiological and biochemical functions of this natural polysaccharide in alleviating oxidative stress and apoptotic pathways in rats with DOX-induced cardiotoxicity. Our findings can be further developed and optimized for human trials.

## Material and methods

### Chemicals

Standard sodium alginate was obtained from Sigma-Aldrich Co. (Cat. No. A1112) (St. Louis, MO, USA). Doxorubicin was bought from EBEWE Pharma Co. (Cat. No. A4866). Acetone, methyl alcohol, 2,2-diphenyl-1-picrylhydrazyl (DPPH^·^), gallic acid, ascorbic acid, chloroform, and ethyl alcohol absolute were of high analytical grade and purchased from Merck Co. (Kenilworth, NJ, USA). Hydrochloric acid (34%), sodium carbonate, and sodium hydroxide were obtained from Abou Zaabal Company for Chemicals, Cairo, Egypt.

### Seaweed sampling and identification

**Fig. 1:**
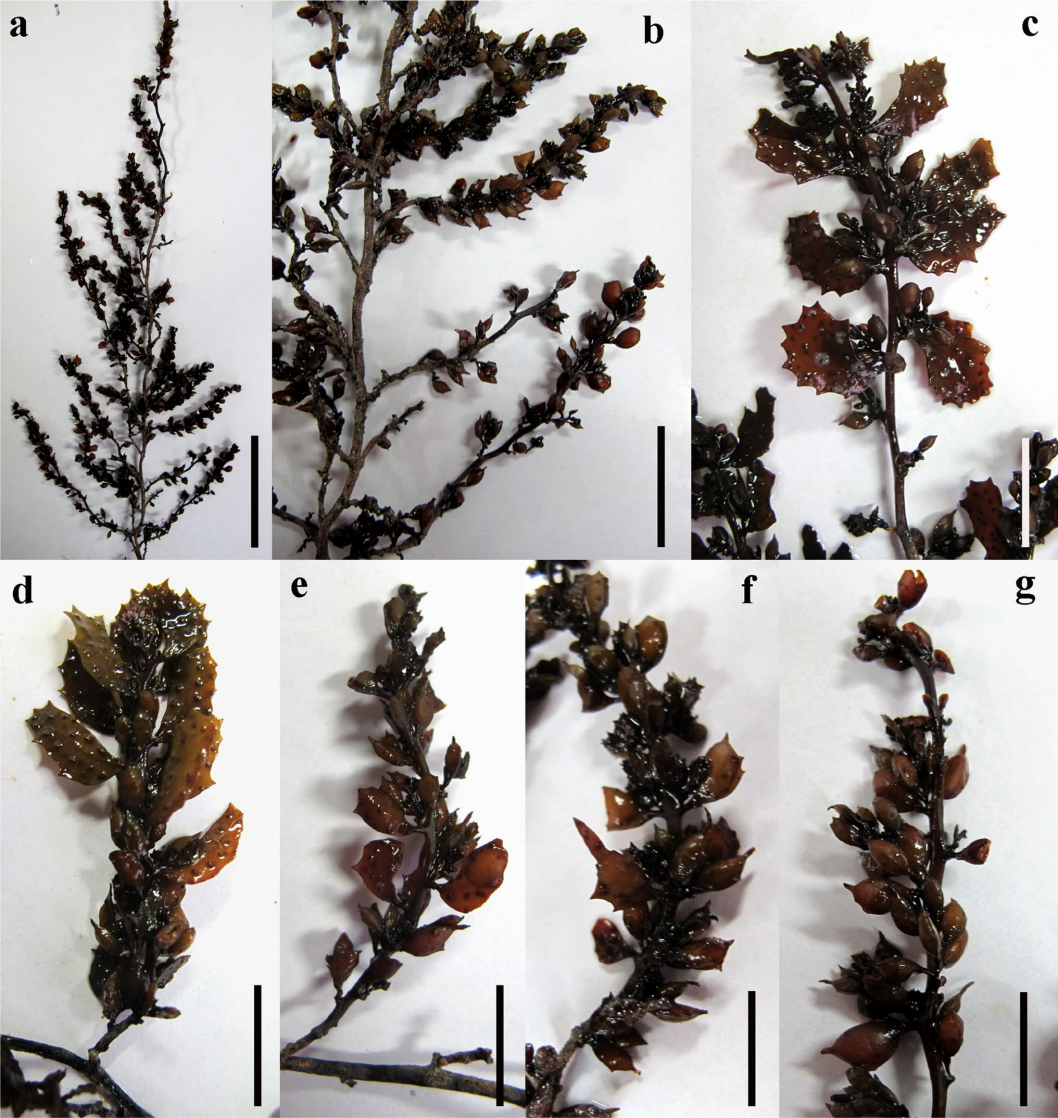
*Sargassum aquifolium* (Turner) C. Agardh morphology. **a**, **b** Habit. **c**, **d** Leaves. **e**–**g** Vesicles. Scale bars: 5 cm (**a**); 2 cm (**b**–**g**)

Thalli of *Sargassum aquifolium* (Turner) C. Agardh were sampled on October 26, 2017 from the littoral zone of rocky shorelines in Hurghada city (27° 15′ 58.45″ N, 33°48′ 57.09″ E), the Red Sea coast of Egypt (Fig. 1). Thalli were washed thoroughly in the field to remove any epiphytes or sand particles, stored in sterile clean plastic bottles, and then transported refrigerated to the laboratory in an ice-box. In the laboratory, the *S. aquifolium* specimens were washed again using tap and distilled waters to be completely free from any salts, sand particles, and debris, and then were shade-air dried at room temperature. Using an electric blender, the dried seaweed specimens were homogenized into a fine powder and then stored in sterile clean plastic bags until needed. *S. aquifolium* specimens were morphologically identified on the basis of the taxonomic classification systems adopted by Mattio et al. (2009) and Krishnamurthy and Ezhili (2013).

### Extraction and purification of sodium alginate

**Fig. 2:**
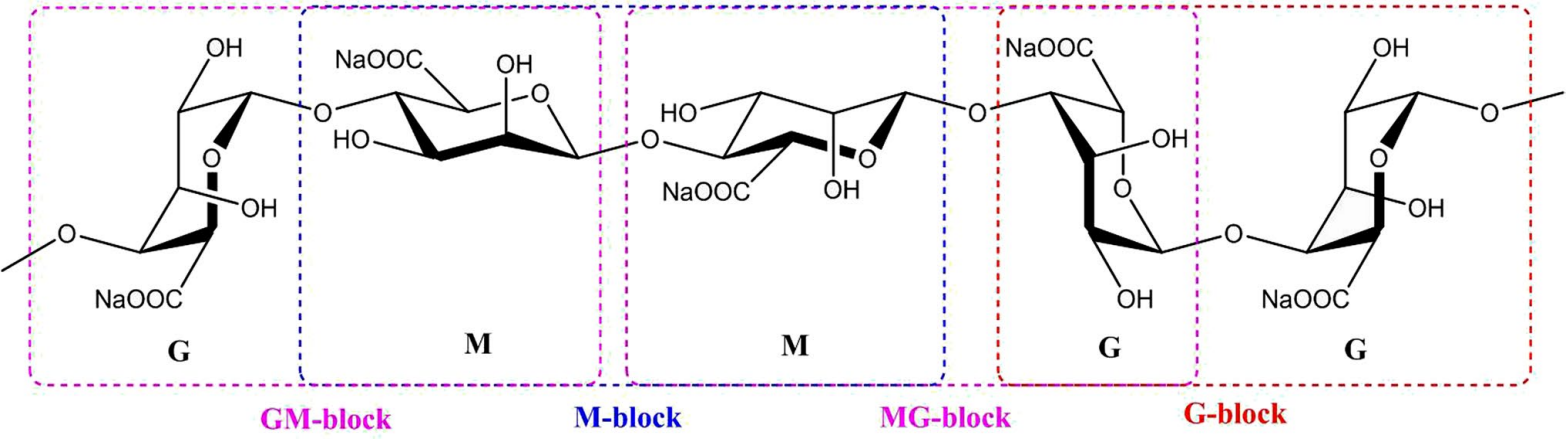
Chemical structure of sodium alginate polymer (after Łabowska et al. 2019)

Purified sodium alginate (Fig. 2) was extracted from the *Sargassum aquifolium* powder following the protocol by Sellimi et al. (2015). Fifty grams of the algal powder was depigmented by treating it twice with acetone–methanol (7:3, 500 ml). Then, it was defatted twice using 300 ml chloroform under constant stirring (250 rpm) for 24 h at 30 °C and air-dried to obtain 45 g of algal powder. Dried algal powder (25 g) was treated with 500 ml of 0.1 M HCl (twice) at approximately pH 2 for 2 h at 60 °C under constant stirring (250 rpm) to demineralize the powder. After centrifugation (5000 rpm, 15 min, 4 °C), the supernatant was removed, and the residue was washed with distilled water. It was then treated with 3% Na_2_CO_3_ (pH 11) at 60 °C for 2 h under constant stirring to solubilize the alginate into its sodium salt form. The supernatant was collected and precipitated with 2 volumes of absolute ethanol. This precipitate was suspended in distilled water and acidified with 2N HCl to pH < 3 to precipitate alginic acid. The formed precipitate was separated, suspended in distilled water, and neutralized using an aqueous 1N NaOH solution (pH 7.5). Finally, the sodium alginate was purified using absolute ethanol in a second precipitation step. The obtained precipitate was dissolved in distilled water and lyophilized to yield pure sodium alginate powder.

### Preparation of the thermally treated sodium alginate

Aqueous solution of the extracted sodium alginate (3%, w/v) was prepared using deionized water. Alginate solution was autoclaved for 6 h at 121 °C and 1.2 bar to obtain the TTSA.

### Chemical characterization of the thermally treated sodium alginate

#### UV–visible spectroscopy

UV–visible spectroscopy of stranded and extracted TTSA was performed at 25 °C by spectrophotometer in 200–400 nm range.

#### Fourier-transform infrared spectroscopy

Fourier-transform infrared spectroscopy (FTIR) spectrometer (Nicolet 550 magna-IR spectrometer) at the wavelength region between 4000 and 400 cm^–1^ was used for obtaining FTIR spectra of the standard and extracted TTSA. These spectra were recorded at the Central Laboratory of Faculty of Science, Ain Shams University. Samples of TTSA were mixed with KBr (1:20 w/w) and were prepared as a disc. The spectra obtained were the result of more than 4 scans with a spectrophotometer resolution of 4 cm^–1^.

Standard alginate: (υ, cm^–1^) 3455 (OH), 2933 (CH aliphatic), 1618 (COO^–^, asymm.), 1417, 1385 (COO^–^, symm.), 1124, 1093, 1030 (C-O), 946, 904, 875, 814, 781 (anomeric sugar residues). Extracted TTSA: (υ, cm^–1^) 3469, 3344 (OH), 2932 (CH aliphatic), 1608 (COO^–^, asymm.), 1417, 1386, 1303 (COO^–^, symm.), 1125, 1096, 1032 (C–O), 949, 889, 820, 782 (anomeric sugar residues).

#### Nuclear magnetic resonance spectroscopy

The nuclear magnetic resonance spectroscopy (^1^H-NMR) spectra of the standard and extracted TTSA were measured on Varian Gemini 300 MHz spectrometer, with chemical shift (δ) expressed in ppm downfield with tetramethylsilane as initial standard, in deuterium water D_2_O and coupling constants J in Hz at the Central Laboratory of Faculty of Science, Cairo University, M-1 and G-1.

Standard alginate: (300 MHz, D_2_O, δ, ppm): 3.84, 3.99, 4.06, 4.10 (m, H-2, H-3 and H-4), 4.26 (s, H-5, GG), 4.55, 4.59 (m, H-1, M; H-5, GM), 5.12 (s, H-1, G). Extracted TTSA: 3.98, 3.95, 4.41 (m, H-2, H-3 and H-4), 4.15 (s, H-5, GG), 4.50 (s, H-1, M; H-5, GM), 5.07 (s, H-1, G).

### Evaluation of antioxidant properties of the thermally treated sodium alginate

We determined the total antioxidant capacity (TAC), DPPH^·^ radical scavenging activity, and ferric reducing antioxidant power assay (FRAP) of TTSA, and untreated alginate from *Sargassum aquifolium* to compare their antioxidant potentials. TAC was assessed following the method described by Prieto et al. (1999). The absorbance was measured at 695 nm using UnicumUV-300UV/Vis spectrophotometer, and TAC was expressed in terms of ascorbic acid equivalents. DPPH^·^ assay was evaluated following the protocol by Cheng et al. (2006). The absorbance was measured at 515 nm, and the percentage inhibition activity was calculated. FRAP was performed according to the method by Oyaizu (1986). The absorbance was measured at 700 nm using gallic acid as the standard. All the measurements were done in triplicate. The data were expressed as mean ± standard error.

### Experimental animals

Sixty adult male Swiss albino rats weighing 150–200 g were obtained from the Egyptian National Research Center breeding unit. The animals were housed in steel mesh cages (5 rats/cage), supplied with a standard pellet diet and water in the animal house of the Pharmacology Department, Faculty of Medicine, Al-Azhar University. The study was conformed to the Guide for the Care and Use of Laboratory Animals published by the National Institutes of Health (No. 85:23, 1996) and also in compliance with the principles and guidelines of the Ethical Committee at the Faculty of Science, Ain Shams University, Cairo, Egypt.

### Experimental design

Sixty rats were randomly divided equally into six groups. The experiment lasted for a week. The doses were selected based on previous studies by Desai et al. (2013) and Guo et al. (2016) as follows:

- *GI (normal control)*: normal healthy animals received oral saline.
- *GII (DOX)*: animals injected intraperitoneally with doxorubicin (DOX) at a dose of 15 mg/kg b.w. (based on Desai et al. 2013) for a week.
- *GIII (TTSA 400)*: animals only received TTSA at a dose of 400 mg/kg b.w. (based on Guo et al. 2016) orally for a week.
- *GIV (DOX* + *untreated SA 400)*: animals were injected intraperitoneally with DOX (15 mg/kg b.w.) and then orally administered with untreated “raw” sodium alginate extract (400 mg/kg b.w.) for a week.
- *GV (DOX* + *TTSA 200)*: animals were injected intraperitoneally with DOX (15 mg/kg b.w.) accompanied by an oral protective dose of TTSA (200 mg/kg b.w) for a week.
- *GVI (DOX* + *TTSA 400)*: animals were injected intraperitoneally with DOX (15 mg/kg b.w.) accompanied by an oral protective dose of TTSA (400 mg/ kg b.w.) for a week.

Blood samples were drawn from the abdominal aorta of each rat and then transferred into a BD Microtainer Blood Collection Tube (BD Life Sciences, Franklin Lakes, NJ, USA). The sera were separated by centrifuging the blood samples at 3000 × *g* for 15 min and stored at − 80 °C for subsequent biochemical testing. The animals were euthanized using cervical dislocation immediately after blood sampling. The cardiac organs of each rat were immediately removed, washed in ice-cold saline, dried, and weighed for additional biochemical investigations. For histopathological evaluation, the cardiac tissue samples were preserved in neutral buffered formalin (10%).

### Biochemical analyses

Serum activity of creatine kinase-MB (CK-MB, EC 2.7.3.2) (Cat. No. 239002) and aspartate transaminase (AST, EC 2.6.1.1) (Cat. No. AS 1061) enzymes were assessed colorimetric by Hitachi™ double beam spectrophotometer using commercially available kits from Spinreact Co. (Carr. de Sta., Coloma, Spain) following the manufacturer’s instructions. Heart superoxide dismutase (SOD, EC 1.15.1.1) (Cat. No. SD 2521) and catalase (CAT, EC 1.11.1.6) (Cat. No. CA 25 17) enzyme activities were determined using commercially available kits from Abcam Co. (Cambridge, UK) using the manufacturer’s instructions. The serum pro-apoptotic caspase-3 levels were estimated using rat CASP-3 ELISA Kit (Cat. No. MBS700575, MyBioSource Co., San Diego, California, USA) based on the quantitative sandwich enzyme immunoassay technique.

### Evaluating MAPK-1 and iNOS genes expression in the heart tissues using quantitative real-time polymerase chain reaction

Total RNA was extracted from the rat hearts using the QIAGEN tissue extraction kit (Cat. No. K0731, QIAGEN, USA) following the manufacturer’s instructions. Quantitative real-time polymerase chain reaction (qRT-PCR) amplification and analysis were conducted using Applied Biosystems with software version 3.1 (StepOne™, USA). The qRT-PCR assay was optimized using the primer sets (Table 1) at a denaturation temperature of 94 °C, annealing temperature of 55 °C, and extension temperature of 72 °C. The complementary DNAs were prepared to determine the relative expression of MAPK-1 and iNOS using GAPDH as an internal control. Ultrapure distilled water was used as the non-template control to validate the absence of DNA contamination. The MAPK-1 and iNOS mRNA expression levels were measured relative to GAPDH following the manufacturer’s protocol. The expression level of the target genes was normalized to GAPDH and expressed as fold changes compared with the control using ΔΔCT method (Livak and Schmittgen 2001).

**Table 1:** Primers sequences of the studied genes

Genes	Primers sequences
MAPK-1	F: 5′-GAC TGA TGC TCT GGG TGA CTG-3′ R: 5′-TTG GAC ATC TGT CCT GCA CT-3′
iNOS	F: 5′-AGA AAC TTC CAG GGG CAA GC-3′ R: 5′-TCC TCA GGC TTG GGT CTT GT-3′
GAPDH	F: 5′-CAAGGTCATCCATGACAACTTTG-3′ R: 5′-GTCCACCACCCTGTTGCTGTAG -3′

*F* forward primer, *R* reverse primer

### Determination of MAPK-1 and p53 by western blotting

Heart tissue proteins were extracted using Trizol reagent, and their concentrations were estimated using the Bradford assay technique. Using 10% SDS–polyacrylamide gel electrophoresis, 20 µg of proteins per lane was electrophoretically separated and transferred onto polyvinylidene difluoride membranes. Then, the membranes were blocked for 2 h at room temperature using a solution containing 5% skimmed dried milk, 10 mM Tris–Cl (pH 7.5), 100 mM NaCl, and 0.1% Tween-20. The membranes were incubated at 4 °C overnight with the selected primary antibodies (MAPK-1, p53, and GAPDH). After incubating with the secondary anti-rabbit monoclonal antibody conjugated to horseradish peroxidase for 2 h at room temperature, the membranes were washed four times with 10 mM Tris–Cl (pH 7.5), 100 mM NaCl, and 0.1% Tween-20 at room temperature. Chemiluminescence was detected using the Amersham detection kit (Cat. No. 170–5060, Thermo Fisher Scientific Co., Massachusetts, USA) following the manufacturer’s protocol. The amount of protein was quantified using densitometric analysis with the Bio-Rad software (Bio-Rad, California, USA). The results were expressed as arbitrary units after normalization with GAPDH protein.

### Histopathological assessment

After the rats were euthanized, the heart tissues were excised, rinsed in a pre-cooled saline solution, and then cut into thin slices. After fixing for 24 h in neutral buffered formalin (10%), the final heart sections were stained with hematoxylin and eosin and observed under a light microscope for histopathological analysis following the standard procedures described by Bancroft et al. (1996). The heart sections were scored blindly and subsamples of heart tissues (one-third of samples scored) was re-scored blindly by a second examinator. Histopathological changes in heart tissues, related to muscle inflammation, were scored on a continuous visual analog scale (ranging from 0 to 3) as described by Mikalsen et al. (2012).

### Statistical analysis

Statistical analysis was conducted using IBM Statistical Package for Social Science (SPSS) software, version 23.0 for Windows (SPSS® Chicago, USA). Results were expressed using descriptive statistics and Mann–Whitney test. A probability of *P* ≤ 0.05 was significant, while *P* > 0.05 was statistically non-significant.

## Results

### Chemical characterization of sodium alginate extracted from Sargassum aquifolium

#### UV–visible spectroscopy

**Fig. 3:**
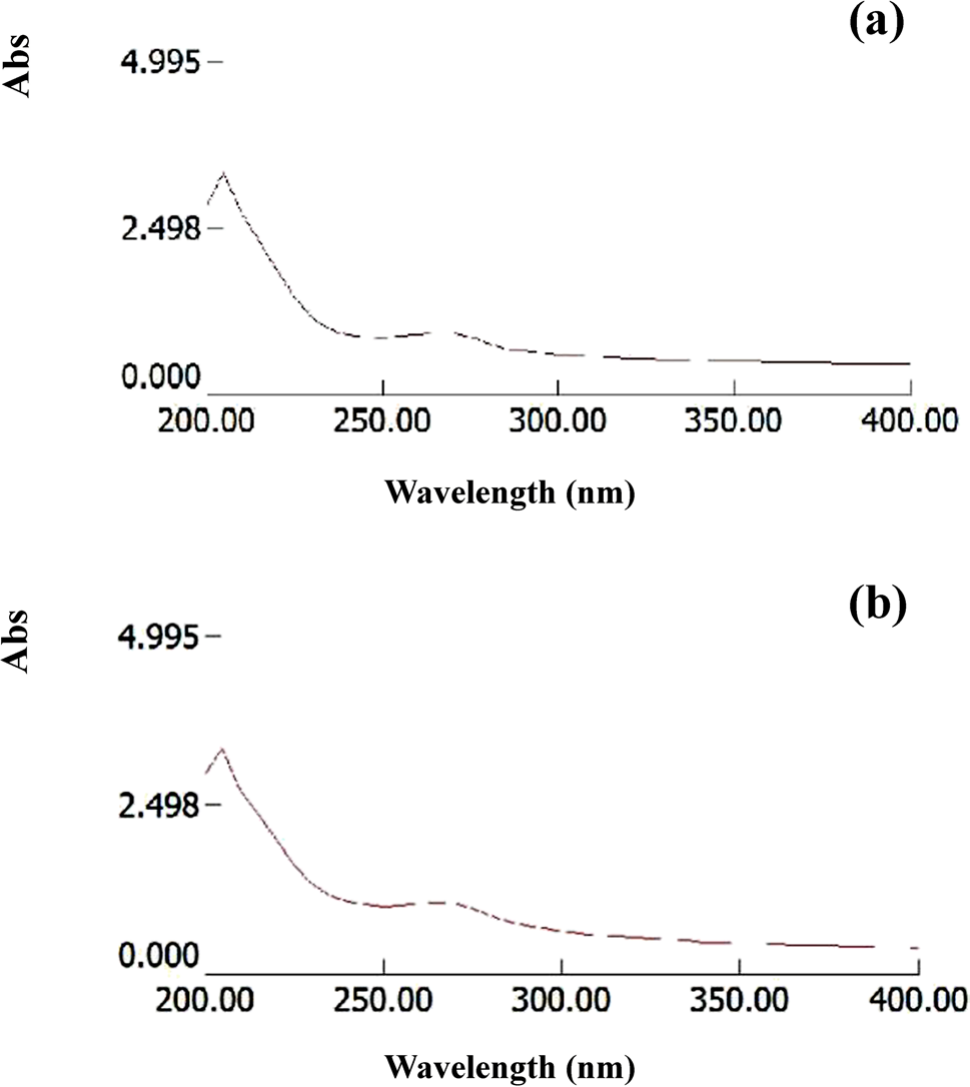
UV spectra of sodium alginates. **a** The standard sodium alginate. **b** The thermally treated sodium alginate (TTSA) derived from *Sargassum aquifolium*

The features of commercial sodium alginate and TTSA were examined by UV–visible spectroscopy. Figure 3 depicts the UV spectra of the standard alginate and TTSA showing a higher absorption band at approximately λ = 265 nm and increased peak intensity for the latter.

#### Fourier-transform infrared spectroscopy

**Fig. 4:**
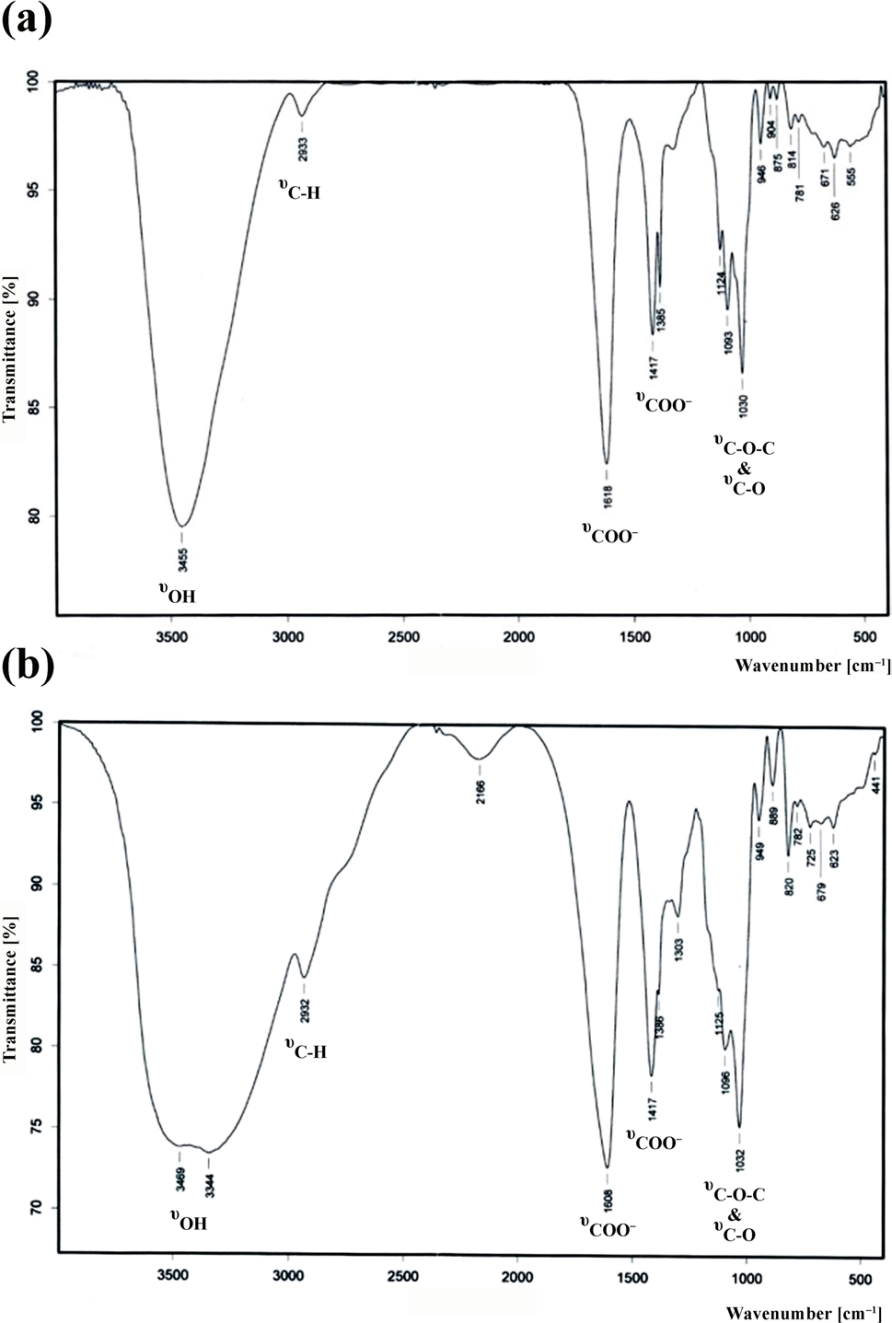
FTIR spectra of sodium alginates. **a** The standard sodium alginate. **b** The thermally treated sodium alginate (TTSA) derived from *Sargassum aquifolium*

The FTIR spectra of the standard sodium alginate and TTSA were placed within the wavelength region of 4000 to 400 cm^–1^ (Fig. 4). The spectra indicate no change in the overall spectral pattern without additional bands. However, minor differences can be observed in the height and shape of certain absorption bands. The spectra of the sodium alginate showed a broad band in the region 3200–3500 cm^−1^, centered at 3455 cm^−1^, characteristic of the H-bonded hydroxyl groups (OH). The band at 2933 cm^−1^ represented the C–H stretching vibration. The bands at ~ 1610 cm^−1^ and 1385 and 1417 cm^−1^ are assigned to carboxylate groups’ asymmetric and symmetric stretching vibration (COO^−^), respectively. The resulting bands placed within 1030 and 1160 cm^−1^ correspond to the glycosidic linkage (C–O–C) and C–O bonds, respectively. The region 950–750 cm^−1^ is assigned to the anomeric uronic acid residues.

#### Nuclear magnetic resonance spectroscopy

**Fig. 5:**
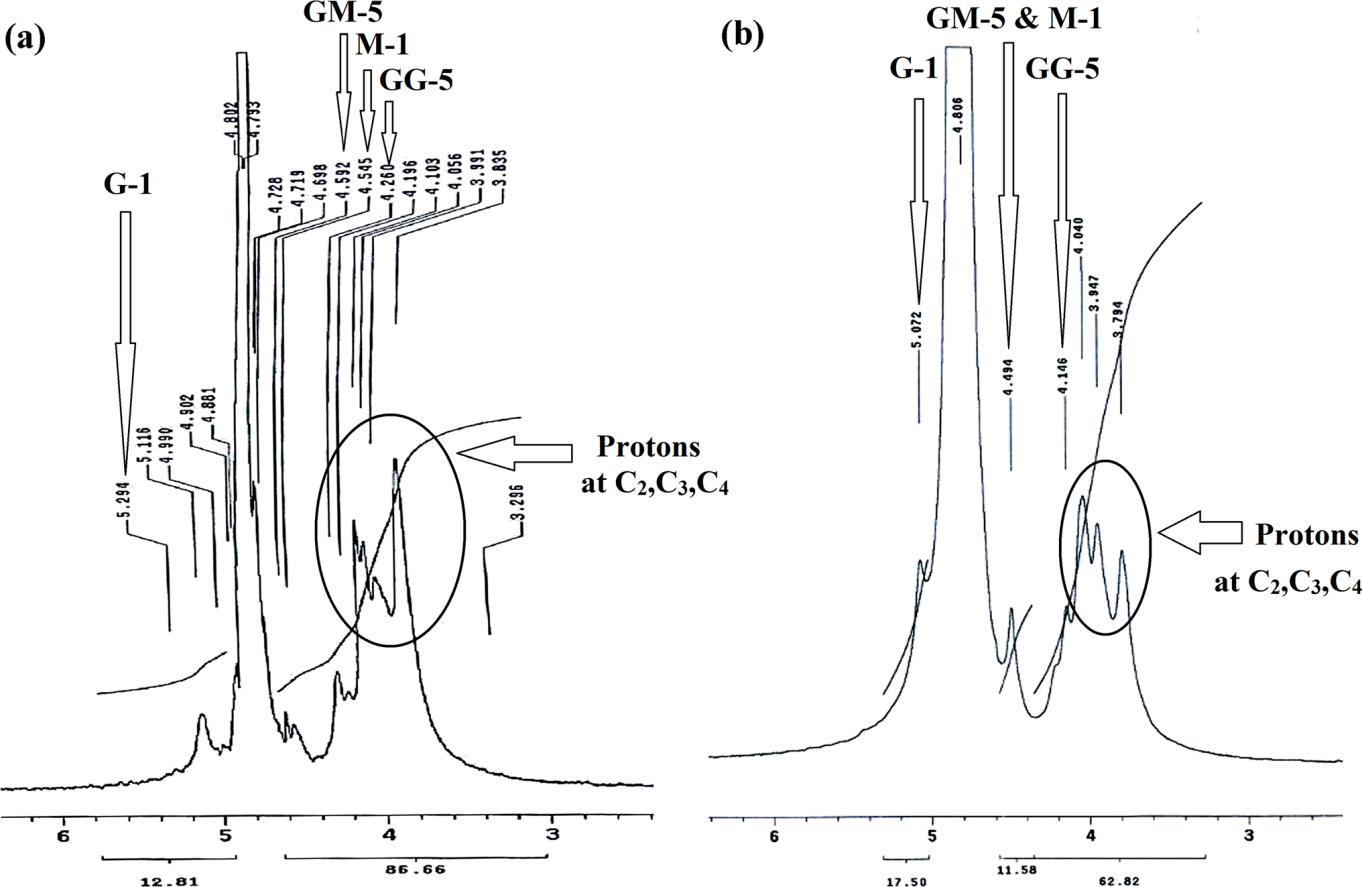
^1^H-NMR spectrum of sodium alginates. **a** The standard sodium alginate. **b** The thermally treated sodium alginate (TTSA) extracted from *Sargassum aquifolium*

The distribution and composition of M and G units in the alginate structure can be determined from the ^1^H-NMR spectroscopy (Davis et al. 2003). The spectra of two alginate samples showed three significant peaks for the protons of guluronate units at C-5 in the GG block (GG-5), β-mannuronate units at C-1 (M-1) + guluronate units at C-5 in the GM block (GM-5), and finally α-guluronate units at C-1 (G-1) in the region 4.2–5.2 ppm for both the standard sodium alginate (Fig. 5a) and TTSA (Fig. 5b). They also displayed peaks at 3.8–4.2 ppm region for protons at C-2, C-3, and C-4.

### Antioxidant characterization

**Fig. 6:**
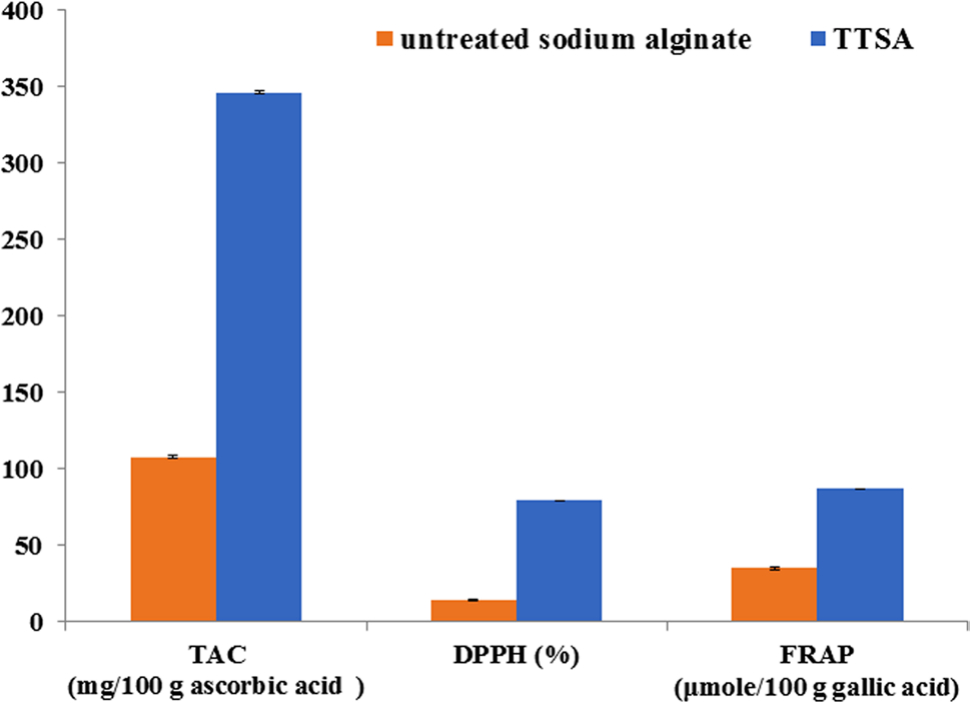
Antioxidant characterization of the thermally treated sodium alginate (TTSA) and the untreated alginate extracted from *Sargassum aquifolium* (mean ± SE)

TTSA exhibited higher antioxidant profiling than the untreated alginate, as shown by the TAC concentration values (345.86 mg/100 g ascorbic acid *vs.* 107.74 mg/100 g ascorbic acid), DPPH^·^ (79.1% *vs.* 14.15%), and FRAP (86.74 μmol/100 g gallic acid *vs.* 35.14 μmol/100 g gallic acid) (Fig. 6).

### Serum levels of biochemical markers

The data in Table 2, Figs. S1, and S2 revealed that the levels of both CK-MB and AST were significantly (*P* = 0.004) higher in the DOX-treated rats than in the control. However, these enzyme levels were significantly decreased after treatment with 200 mg/kg or 400 mg/kg TTSA in the DOX-injected rats compared with the untreated DOX-injected rats. Similarly, significantly lower enzyme levels were obtained (*P* = 0.004 and *P* = 0.006 for AST and CK-MB, respectively) in the animal group administered with raw sodium alginate (GIV) compared to the DOX-treated group. Notably, the 400 mg/kg TTSA group (GIII) showed significantly (*P* = 0.004) lower AST and CK-MB levels than the DOX-injected group, without any significant differences compared with the control.

**Table 2:** Statistical data of biochemical biomarkers investigated in all animal groups

Groups		AST (U/l)	CK-MB (U/l)	Caspase-3 (ng/ml)	SOD (U/g)	CAT (U/g)
GI	Median	54	339.65	1.11	26,992.5	44.16
	IQR	52–56.25	264.8–390	0.99–1.55	21,795–29,508.75	26.81–82.02
GII	Median	99.5	935.05	8.7	8332.5	30.41
	IQR	93.25–100.4	562.1–990.3	6.7–10.25	1020 –16,020	30.15–51.2
	*P*-value	*P* = 0.004^a^	*P* = 0.004^a^	*P* = 0.004^a^	*P* = 0.004^a^	*P* = 0.74^a^
GIII	Median	58	404.6	1.075	29,404	101.42
	IQR	54.75–60.75	384.9–455.8	1.02–1.23	24,378.8–31,976	67.5–284.4
	*P*-value	*P* = 0.075^a^ *P* = 0.004^b^	*P* = 0.055^a^ *P* = 0.004^b^	*P* = 0.936^a^ *P* = 0.004^b^	*P* = 0.423^a^ *P* = 0.004^b^	*P* = 0.024^a^ *P* = 0.004^b^
GIV	Median	59	470.45	2.1	29,755	120.52
	IQR	58–65	456.6–513.3	1.6–2.56	27,255–30,255	103.43–133.22
	*P*-value	*P* = 0.035^a^ *P* = 0.004^b^	*P* = 0.004^a^ *P* = 0.006^b^	*P* = 0.016^a^ *P* = 0.004^b^	*P* = 0.19^a^ *P* = 0.003^b^	*P* = 0.004^a^ *P* = 0.004^b^
GV	Median	59.5	449	2.5	30,132	101.08
	IQR	53.8–85	410.6–567.5	2.09–5.18	26,891–31,950	100–102
	*P*-value	*P* = 0.146^a^ *P* = 0.004^b^	*P* = 0.01^a^ *P* = 0.016^b^	*P* = 0.004^a^ *P* = 0.006^b^	*P* = 0.26^a^ *P* = 0.004^b^	*P* = 0.004^a^ *P* = 0.004^b^
GVI	Median	52	347.55	2.75	34,125	108.38
	IQR	50.75–57.25	338.4–466.32	2.25–4.12	2808 –34,125	93.62–141.52
	*P*-value	*P* = 0.421^a^ *P* = 0.004^b^	*P* = 0.336^a^ *P* = 0.004^b^	*P* = 0.004^a^ *P* = 0.004^b^	*P* = 0.009^a^ *P* = 0.003^b^	*P* = 0.004^a^ *P* = 0.004^b^

GI, control; GII, rats only administrated doxorubicin (DOX); GIII, rats only received thermally treated sodium alginate (TTSA) with a dose of 400 mg/kg b.w.; GIV, rats given DOX and 400 mg/kg b.w. raw sodium alginate; GV, rats administrated DOX and 200 mg/kg b.w. TTSA; GVI, rats received DOX and 400 mg/kg b.w. TTSA. Data are expressed as median (25th, 75th quartiles). *P* is significant at values ≤ 0.05

^a^Significance *vs.* the control

^b^Significance *vs.* the DOX-administrated animal group

The expression level of caspase-3 (Table 2 and Fig. S3) was significantly higher in the DOX-injected group (GII) compared to the control group. Contrarily, the anaphylactic groups treated with either raw sodium alginate (GIV) or two different doses of TTSA (GV and GVI) showed significantly lower caspase-3 levels than the DOX-injected group.

The activities of SOD and CAT enzymes were evaluated (Table 2; Figs. S4 and S5) to assess the antioxidant efficacy of the alginates in alleviating oxidative stress. Remarkably, the antioxidant enzyme activities were significantly higher in all the groups treated with the two different doses of TTSA or raw sodium alginate extract than in the control and DOX-injected rats.

### Gene expressions of MAPK-1 and iNOS

**Fig. 7:**
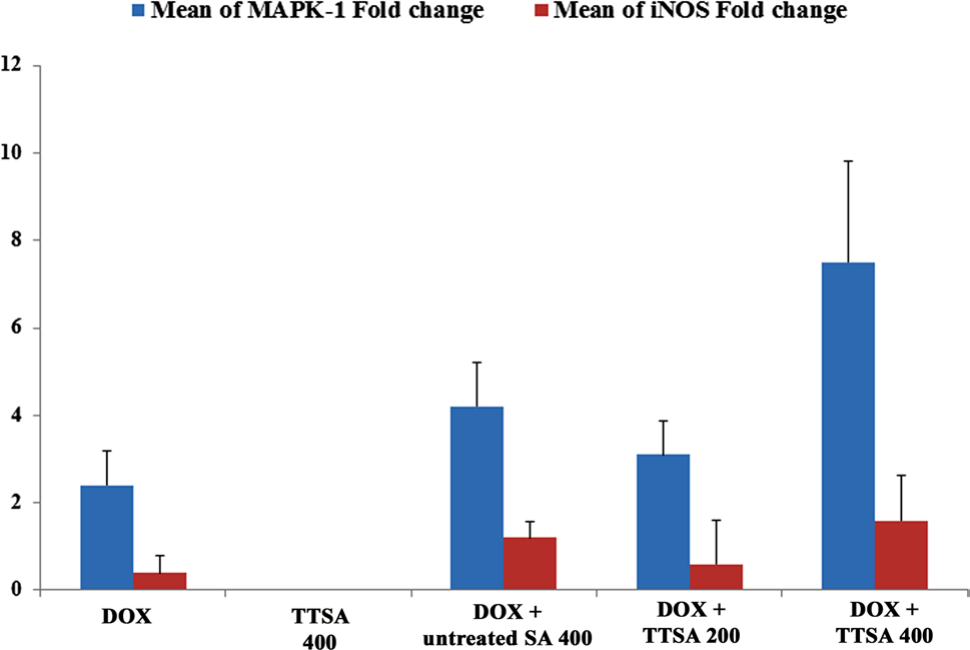
Relative quantification of MAPK-1 and iNOS gene expressions (mean ± SE) in all studied animal groups

As illustrated in Fig. 7, the treatment with the TTSA alone (GIII) did not show any distinctive effect on the expression of the MAPK-1 gene. However, DOX administration significantly upregulated the MAPK-1 gene expression by 2.3-fold compared to the control. This effect was also significantly increased to 3.1- and 7.46-fold changes by the TTSA treatments with both doses of 200 mg/kg (GV) and 400 mg/kg (GVI), respectively. A similar observation was obtained in treatment with the untreated raw sodium alginate extract (400 mg/kg).

Treatments with 400 mg/kg each of TTSA (GVI) and untreated raw alginate (GIV) extracts upregulated the expression of iNOS genes by 1.5- and 1.2-fold, respectively, compared with the control (Fig. 7). However, 200 mg/kg TTSA (GV) treatment resulted in a non-significant upregulation of iNOS gene expression. Administration of 400 mg/kg TTSA (GIII) and DOX (GII) showed a significant decrease in iNOS gene expression.

### Protein expressions of MAPK-1 and p53

**Fig. 8:**
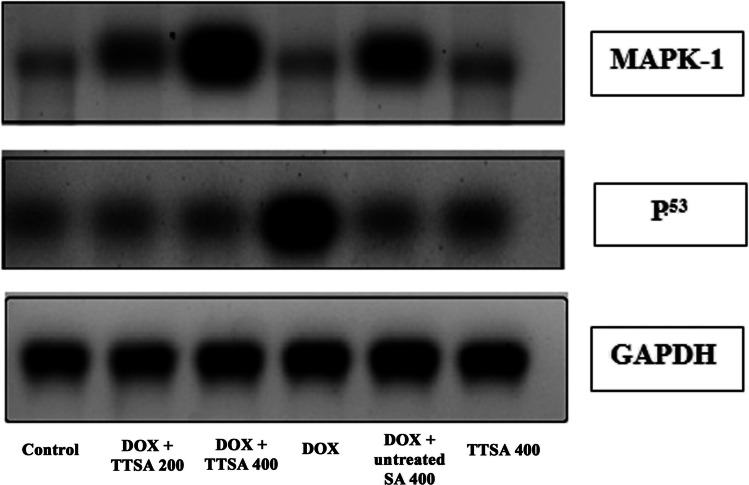
Western blot analyses of MAPK-1 and p53 in all studied animal groups

In the DOX-injected groups treated with 200 and 400 mg/kg TTSA, the MAPK-1 gene expression level (Fig. 8 and Fig. S6) were increased by 3.1- and 7.5-fold, respectively, compared with the 4.2-fold increase in rats administrated with 400 mg/kg of untreated raw sodium alginate (GIV) only and 2.4-fold change in the DOX-injected group (GII). There was no change in the groups administered only with 400 mg/kg TTSA (GIII). The p53 level was significantly increased in the DOX-injected group, while no significant change was seen in all other groups compared with the control (Fig. 8 and Fig. S7).

### Histopathological examination of myocardial tissues

**Fig. 9:**
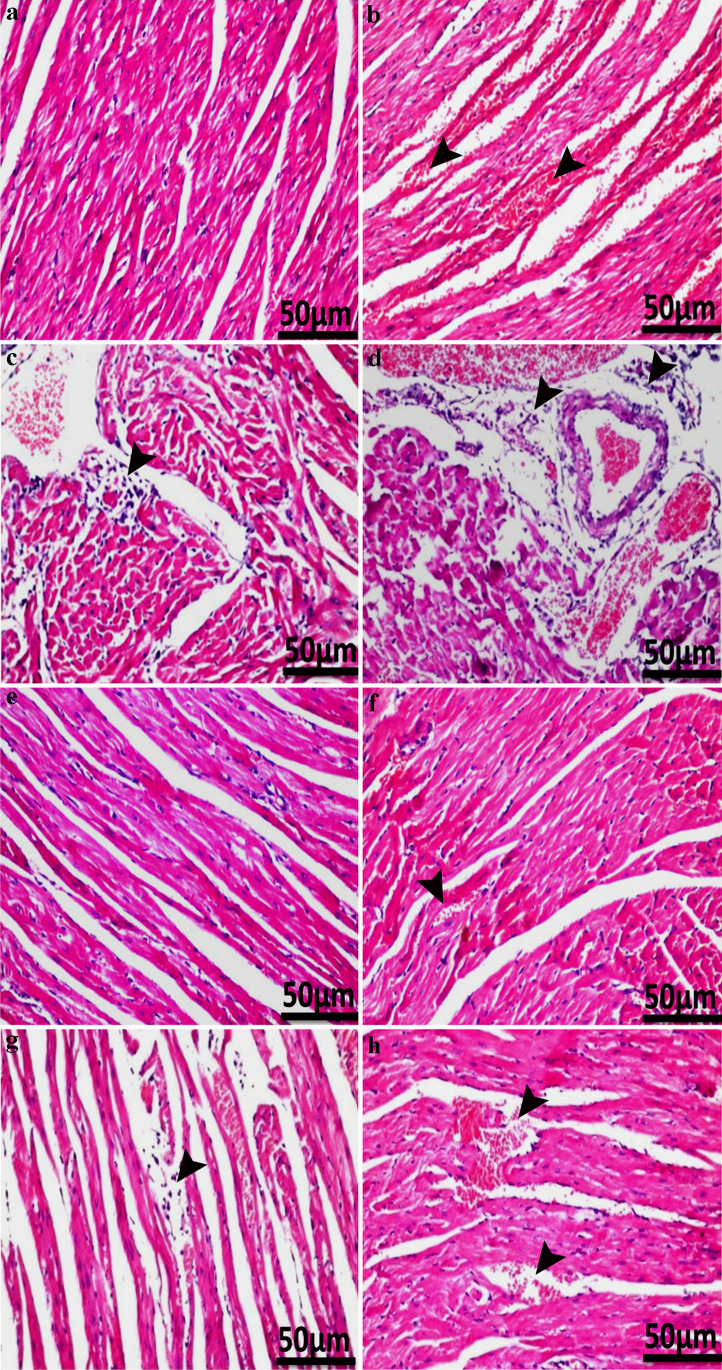
Light microscopy images of the rat heart tissues depicting histopathological treatments in this study. **a** Non-treated control. **b**–**d** Rats only injected with doxorubicin. Note focal hemorrhages (arrowheads) in **b**, inflammatory cells infiltration with edema (arrowhead) in **c**, and inflammatory cells infiltration (arrowheads) in **d**. **e** Normal rats only received the thermally treated sodium alginate (TTSA) with a dose of 400 mg/kg b.w. **f** DOX-administered rats treated with raw sodium alginate (400 mg/kg b.w.). Note inflammatory cells infiltration (arrowhead). **g** DOX-administered rats given 200 mg/kg b.w TTSA. Note weak inflammatory cell infiltration (arrowhead). **h** DOX-administered rats given 400 mg/kg b.w TTSA. Note a few focal hemorrhages (arrowheads). H&E stain used. Scale bars: 50 μm

To examine the efficacy of the untreated raw sodium alginate and the two different doses of TTSA (200 and 400 mg/kg) on attenuating DOX-induced cardiac injuries, the heart sections were prepared, stained with H&E, and visualized using light microscopy. The myocardial bundles in the control group showed normal histological structure (Fig. 9a) while the DOX-injected group showed focal hemorrhages (Fig. 9b) and focal inflammatory cell infiltration within the myocardial bundles (Fig. 9c). Meanwhile, perivascular inflammatory cell infiltration with edema surrounding the dilated and congested blood vessels was seen in the myocardium (Fig. 9d). Treatment with 400 mg/kg of TTSA alone (GIII) did not cause any histopathological alteration in the myocardium (Fig. 9e). Animals administrated with DOX and untreated raw sodium alginate extract (400 mg/kg b.w.) (GIV) exhibited relatively normal histological structure of myocardial tissues compared with the control (Fig. 9f). Furthermore, DOX-injected animals administrated orally with 200 and 400 mg/kg b.w. of TTSA (GV and GVI, respectively) showed edema with few focal inflammatory cell infiltration within the myocardial bundles (Fig. 9g) and almost normal myocardial tissues with weakly focal hemorrhages (Fig. 9h), indicating the potential cardiovascular amelioration induced by the TTSA (Table 3).

**Table 3:** The histopathologic lesion scores of cardiac tissues in the animal groups

Lesions	GI	GII	GIII	GIV	GV	GVI
Focal hemorrhages	0	3	0	1	0	1
Inflammatory cell infiltration	0	3	0	0	1	0
Inflammatory cell infiltration with edema	0	3	0	0	0	0

GI, control; GII, rats only administrated doxorubicin (DOX); GIII, rats only received thermally treated sodium alginate (TTSA) with a dose of 400 mg/kg b.w.; GIV, rats given DOX and 400 mg/kg b.w. raw sodium alginate; GV, rats administrated DOX and 200 mg/kg b.w. TTSA; GVI, rats received DOX and 400 mg/kg b.w. TTSA. 0 = none, 1 = mild, 3 = severe

## Discussion

According to global statistics, cancer is the leading cause of morbidity and mortality. Despite the ongoing development in alternative cancer treatment techniques, systemic chemotherapy remains the gold standard. Anthracyclines, particularly DOX, have been the keystone therapeutics for hematological and solid malignancies. Despite their broad-spectrum anticancer properties, anthracyclines have been limited in clinical practice due to their diverse side effects, including cardiovascular complications, particularly in patients requiring dose escalation due to the advanced disease (Zamorano et al. 2016). Anthracycline-mediated cardiomyopathy has been linked to oxidative stress, systemic inflammation, and calcium metabolism issues (Wallace et al. 2020).

While the mechanisms proposed for DOX-induced cardiotoxicity are still controversial, there are a few hypotheses regarding this (Šimůnek et al. 2009; Murabito et al. 2020). For instance, DOX might be oxidized to the unstable metabolite “semiquinone,” which is then transformed back to DOX, releasing numerous reactive oxygen species (ROS). ROS triggers different oxidative stress patterns, lipid peroxidation, and membrane and DNA damage (Menna et al. 2012; Jones and Dass 2022). They can also initiate apoptotic cell death pathways (Thorn et al. 2011; Su et al. 2019). Other potential hypotheses for the development of DOX-induced cardiotoxicity include low levels of antioxidants and sulfhydryl groups in the cardiac muscles (Ichikawa et al. 2012; Rawat et al. 2021), suppression of nucleic acid and protein syntheses (Marechal et al. 2011), the release of vasoactive amines (Clementi et al. 2003; Baniahmad et al. 2020), altered adrenergic function (Kwok and Richardson 2002; Rawat et al. 2021), and downregulated expression of cardiac-specific genes (Wallace et al. 2020).

The research on developing innovative formulations from marine resources such as seaweeds has recently gained popularity. Seaweeds are an excellent source of structurally diverse and chemically different bioactive constituents, characterized by numerous pharmaceutical and biomedical applications (Ibrahim et al. 2021; Semaida et al. 2022). In this study, we chemically characterized TTSA, derived from *Sargassum aquifolium*. The formation of bands in the region 250–280 nm in the NMR spectra was attributed to the presence of carbonyl groups. Increased carbonyl band intensity (λ = 265 nm) of the carboxylate groups in the TTSA was due to the increased concentration of sodium alginate, indicating the higher purity of this sample. For the TTSA sample, all the intensity absorption bands were remarkably increased compared with the chemical standard, particularly the bands at ~ 1610 and 3455 cm^–1^ corresponding to the asymmetric carboxylate group and hydroxyl sugar residues, respectively (Fig. 4). This also proved the higher purity of TTSA. This finding is also consistent with the results obtained from the UV–visible spectroscopy (Fig. 3). Moreover, we estimated the M/G ratio of TTSA using FTIR spectra. The ratio of absorption band intensities assigned to the M and G monomers was at approximately 1100 and 1025 cm^−1^, respectively, providing a reasonably accurate estimate of the M/G ratio (Pereira et al. 2003). M/G ratio of < 1 indicates a lower percentage of M units than G units, which corresponds to elastic and flexible alginates. Conversely, alginates with M/G ratio of > 1 have higher percentage of M units and form brittle gels (Murillo-Álvarez and Hernández-Carmona 2007). The M/G ratio of the chemical standard and TTSA were < 1, with a relatively higher ratio for TTSA.

The M/G ratio and the GG fraction were evaluated from the proton area values of the three signals: GG-5, M-1, and G-1 as M/G ratio = M-1/G-1 and GG fraction = GG-5/G-1 (Khajouei et al. 2018; Gomez et al. 2009). The calculations from the integration values showed that the M/G ratio is < 1 for the standard and TTSA, with a higher value for TTSA due to a slight increase in the integration of the M-1 signal. The GG fraction in the TTSA was lower than the standard alginate due to the lower GG-5 signal integration compared with the G-1 signal in the TTSA. Thus, we concluded that the TTSA is purer and more elastic than the standard alginate based on the UV, IR, and ^1^H-NMR results (Figs. 3, 4, 5).

TTSA exhibited better antioxidant activity than untreated polymeric alginate. This enhanced biological activity might be due to the breakdown of the complex polymeric alginate and the formation of low molecular weight oligosaccharides with much more functional hydroxyl (–OH) groups that highly contribute to its antioxidant activity. Consistent with our hypothesis, Falkeborg et al. (2014) and Huang et al. (2021) depolymerized the complex polysaccharide alginate into oligosaccharides using alginate lyase, which displayed better antioxidant activity due to the presence of the conjugated alkene acid structure formed during enzymatic depolymerization. Luan et al. (2009) and Chang et al. (2022) also used radiation to depolymerize alginate and proposed that the general process of radiation-chemical transformation causes glycosidic bond disintegration. Carbonyl groups and double bonds between the C-4 and C-5 residues are formed during this process. We confirmed the carbonyl groups and double bonds between the C-4 and C-5 units using different spectroscopic measurements. This might be the possible underlying mechanism involved in alginate degradation by thermal treatment using autoclaving.

Based on our in vivo findings, we suggest that TTSA is a promising natural supplement with therapeutic effects against DOX-induced cardiotoxicity. Administration of TTSA ameliorated the elevated heart enzymes AST and CK-MB levels. Alginates significantly decreased cardiac enzymes in a dose-dependent manner, consistent with a study by Khotimchenko and Khotimchenko (2004), who emphasized that pretreatment of calcium alginate can inhibit certain blood enzymes and lipid peroxidation products in a dose-dependent manner before carbon tetrachloride (CCl4) administration. DOX intake and cardiotoxicity generated free radicals and ROS (Lee et al. 1991; Saleh et al. 2022), elevating AST and CK-MB enzyme levels. However, TTSA could potentially attenuate free radical chain reactions at early stages, thus inhibiting the damage induced by excess free radicals induced by DOX. This is consistent with the study by Zhao et al. (2012), who emphasized that low molecular weight alginates, composed of guluronic and mannuronic acids, exhibited better antioxidant activity on superoxide and hydroxyl radicals. Several previous studies have shown the antioxidant and anti-inflammatory activities of alginates. Alginates can scavenge excess ROS free radicals, nitric oxide, prostaglandin E2, and the cyclooxygenase COX-2 (Elbayomi et al. 2021; Pan et al. 2021; Kaidi et al. 2022). Recently, Liu et al. (2019) showed that alginate could stimulate monocytes to secrete anti-inflammatory cytokines, which might be a possible reason behind its antioxidant effect against DOX-induced toxicity.

Mitogen-activated protein kinase (MAPK) signaling pathways are critical intermediates of oxidative stress-induced apoptosis. Extracellular signal-regulated kinases (ERKs), p38-MAPK, and c-Jun NH2-terminal kinases/stress-activated protein kinases (JNKS/SAPKs) are the three primary MAPK cascades. MAPKs are essential for the cardiac responses to pathological conditions in rats. ERKs are activated by cytokines in the cardiovascular system, mediating cell survival and providing cytoprotection. p38-MAPKs and JNKs are activated by cellular stressors such as oxidative stress and might be linked to cardiomyocyte apoptosis and cardiac pathologies (Šimončíková et al. 2008; Meijles et al. 2020). In this study, we observed significantly higher gene expression levels of MAPK-1 and iNOS in both the TTSA and untreated raw alginate groups compared with the DOX-injected rats (Fig. 7). These observations coincide with the hypothesis proposed by Šimončíková et al. (2008), who stated that several cardioprotective substances are generated in response to DOX administration. Furthermore, they observed elevated activation of ERKs, suggesting the possible adaptive-physiological response of the myocardial tissues to DOX treatment (Šimončíková et al. 2008). The functional role of the ERK pathway in regulating responses to DOX has also been discussed in cultured rat neonatal cardiomyocytes. ERK activation typically enables cell survival, implying that the ERK activation might be implicated in the adaptive responses toward DOX-induced myocardial damage. Accordingly, the activation of ERK1/2 and Akt in adult rat myocytes served as a salvage pathway against the detrimental effects of DOX (Sawyer et al. 2002). Furthermore, MAPK pathways are crucial in regulating DNA damage–induced cell death (Takimoto and Kass 2007; Rezatabar et al. 2019). In this context, our results are consistent with the findings of Lou et al. (2005), who showed that ERK1/2 is activated early, transiently, and downregulated during heart failure. This early upregulation might be an adaptive-protective response.p53 is a tumor suppressor gene responsible for cell cycle and apoptosis. Several previous studies have shown that p53 is activated in oxidation-damaged cells by regulating apoptosis. p53-regulated cell death pathways have been linked to acute DOX-induced cardiotoxicity, leading to cardiac dysfunction and death (Fang et al. 2019). Our results showed that p53 levels were significantly increased in the DOX-injected group, while the changes in all other groups were non-significant compared to the control (Fig. 8). This is consistent with previous observations by Zhu et al. (2009), who highlighted that DOX-induced cardiomyocyte apoptosis is directly proportional to the elevated expression of the p53 tumor suppressor protein. Mancilla et al. (2020) also showed that the p53 expression is increased in primary cardiac fibroblasts after DOX exposure. Liu et al. (2004) reported a significant reduction in DOX-associated cardiac dysfunction and cardiomyocyte apoptosis upon inhibition of p53 nuclear translocation. Conversely, Li et al. (2019) concluded that p53 might occasionally have a cardiac protective effect against a chronic form of DOX cardiotoxicity. However, it is well established that this gene biomarker regulates the programmed cell death pathway in acute DOX cardiotoxicity. Therefore, we believe that further in-depth studies are still required to clarify this. Lastly, Feng et al. (2021) highlighted that in vivo treatment with alginate oligosaccharides in C57BL/6 J mice might decrease p53 protein synthesis in a dose-dependently manner, along with significantly increasing the endogenous antioxidant enzymes SOD1, SOD2, and CAT. Based on our observations, we confirm that TTSA, particularly at a dose of 400 mg/kg b.w., has potential cardioprotective effects and can be considered a natural, safe, antioxidant, and anti-apoptotic biopolymer for treating DOX-induced cardiotoxicity and apoptotic pathways.

We showed that treatment with 400 mg/kg b.w. of TTSA significantly ameliorated DOX-induced heart tissue injury. This observation was confirmed by the upregulation in the antioxidant enzymes SOD and CAT, decrease in the cardiac biomarkers (CK-MB and AST) levels, and normalization of the caspase-3 level (Table 2). TTSA might putatively exert its cardioprotective efficacy through the following mechanisms: (1) scavenging the free radicals released in the cardiovascular tissues; (2) suppressing cell apoptosis caused by DOX exposure; (3) upregulating the endogenous enzymatic antioxidant pathways to ameliorate the adverse effects of DOX-induced oxidative stress and toxicity. Our observations are consistent with the study by Guo et al. (2016), who confirmed that alginate oligosaccharides might increase the survival rate of DOX-induced mice by attenuating cardiac oxidative stress and suppressing the expression of gp91 and 4-hydroxynonenal. Liu et al. (2021) also clarified that administration of *Sargassum fusiforme*–derived alginates to diabetic mice might attenuate several pathological cascades in the heart, hepatic, and adipose tissues and diminish the oxidative stress paradigms. We observed significant downregulation in the expression of the caspase-3 protein in all the alginate-treated groups, especially the TTSA-treated ones, compared with the DOX-injected group (Table 2). This observation is consistent with the results by Guo et al. (2013) and Yarmohammadi et al. (2021), who noticed that pretreatment with alginate oligosaccharides attenuated caspase-12 cleavage induced by DOX administration, thus, ameliorating myocardial apoptosis and cardiac dysfunction.

## Conclusion

Based on our findings, we concluded that the antioxidant properties of sodium alginate, derived from *Sargassum aquifolium*, are enhanced by thermal treatment. Furthermore, its pharmaceutical efficacy as a potential cardioprotective supplement was also improved, as shown by increased expression of cardioprotective mediators, downregulated pro-apoptotic markers, and normalized cellular redox potential. TTSA, specifically at a dose of 400 mg/kg b.w., might act as a prophylactic in the treatment of DOX-linked cardiotoxicity and apoptosis.

## Supplementary Information

ESM 1 Box–plot for concentrations of the enzyme aspartate transaminase (AST) in all investigated animal groups.(PNG 31 kb)

High resolution image (TIF 94 kb)

ESM 2 Box–plot for concentrations of the enzyme creatine kinase-MB (CK-MB) in all investigated animal groups.(PNG 32 kb)

High resolution image (TIF 99 kb)

ESM 3 Box–plot for concentrations of the pro-apoptotic caspase-3 in all investigated animal groups.(PNG 31 kb)

High resolution image (TIF 83 kb)

ESM 4 Box–plot for concentrations of the enzyme superoxide dismutase (SOD) in all investigated animal groups.(PNG 9 kb)

High resolution image (TIF 66 kb)

ESM 5 Box–plot for concentrations of the enzyme catalase (CAT) in all investigated animal groups.(PNG 11 kb)

High resolution image (TIF 69 kb)

ESM 6 (DOCX 520 kb)

ESM 7 (DOCX 438 kb)

## Data Availability

Not applicable.
