# Supplementary figures and images for: Seaweed Sargassum aquifolium extract ameliorates cardiotoxicity induced by doxorubicin in rats
Source: Environ Sci Pollut Res Int. 2023 Mar 28;30(20):58226–42. doi: 10.1007/s11356-023-26259-z (PMC10163098; doi:10.1007/s11356-023-26259-z)

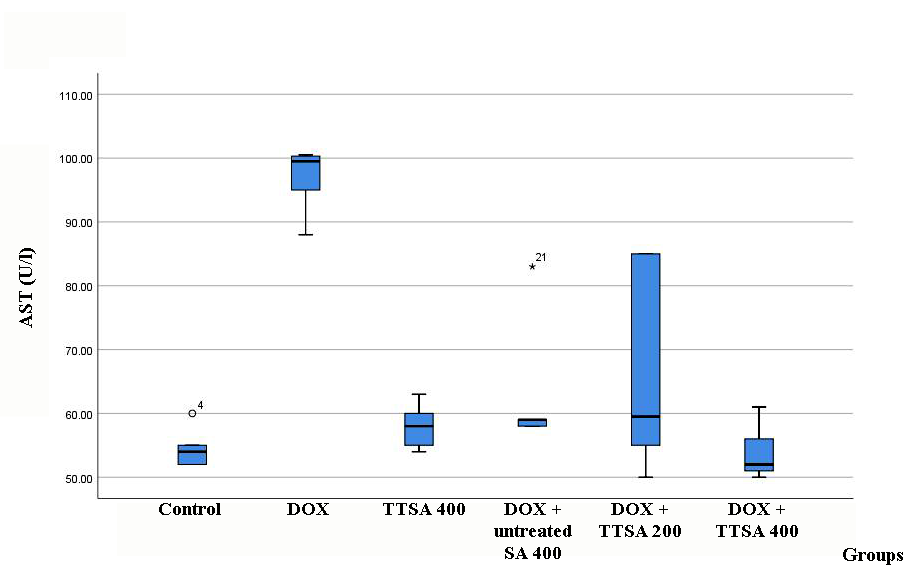

Supplement: Supplementary file 1 — Box–plot for concentrations of the enzyme aspartate transaminase (AST) in all investigated animal groups.(PNG 31 kb) [file 11356_2023_26259_Fig10_ESM.png]

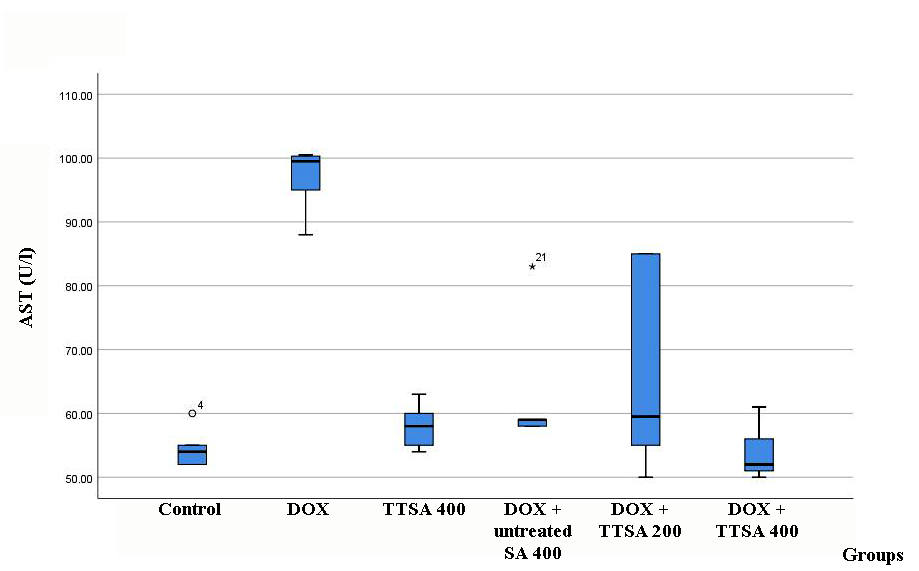

Supplement: Supplementary file 2 — High resolution image (TIF 94 kb) [file 11356_2023_26259_MOESM1_ESM.tif]

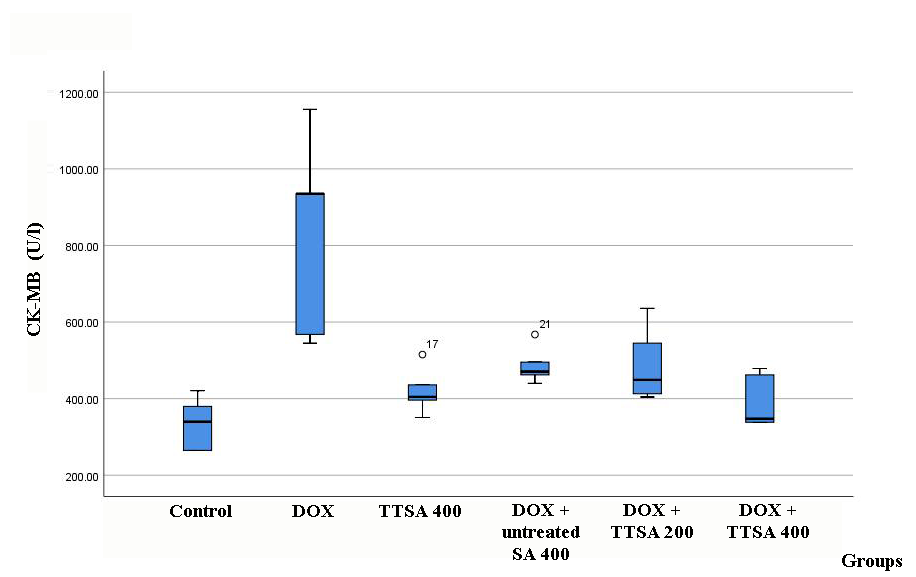

Supplement: Supplementary file 3 — Box–plot for concentrations of the enzyme creatine kinase-MB (CK-MB) in all investigated animal groups.(PNG 32 kb) [file 11356_2023_26259_Fig11_ESM.png]

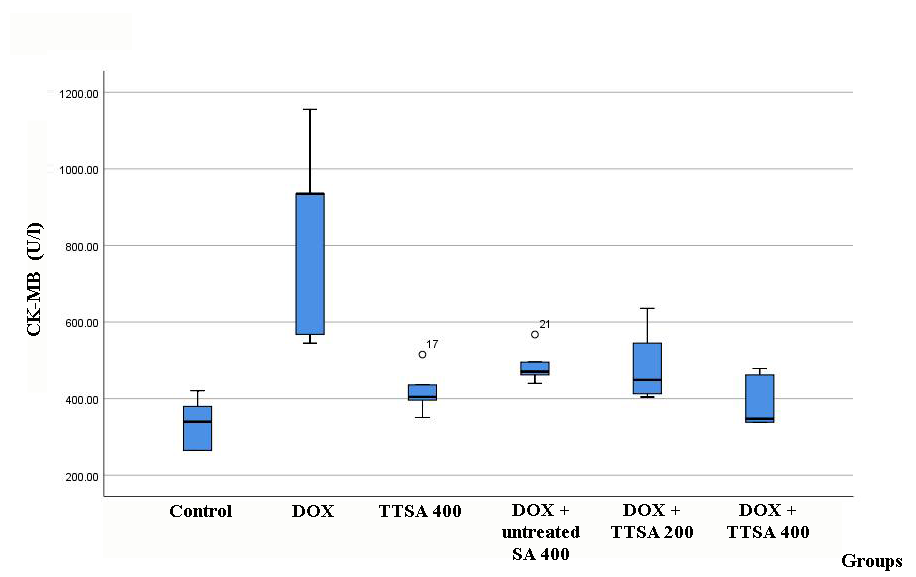

Supplement: Supplementary file 4 — High resolution image (TIF 99 kb) [file 11356_2023_26259_MOESM2_ESM.tif]

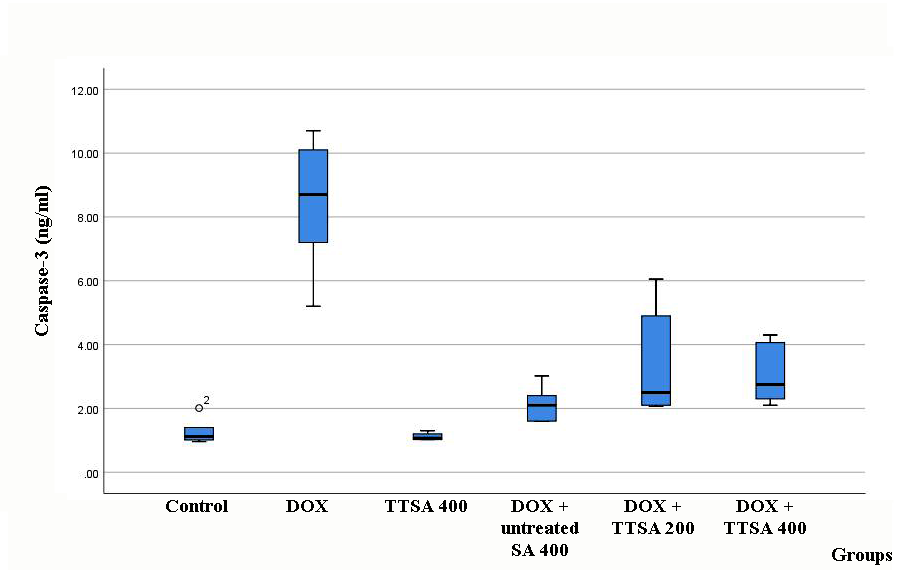

Supplement: Supplementary file 5 — Box–plot for concentrations of the pro-apoptotic caspase-3 in all investigated animal groups.(PNG 31 kb) [file 11356_2023_26259_Fig12_ESM.png]

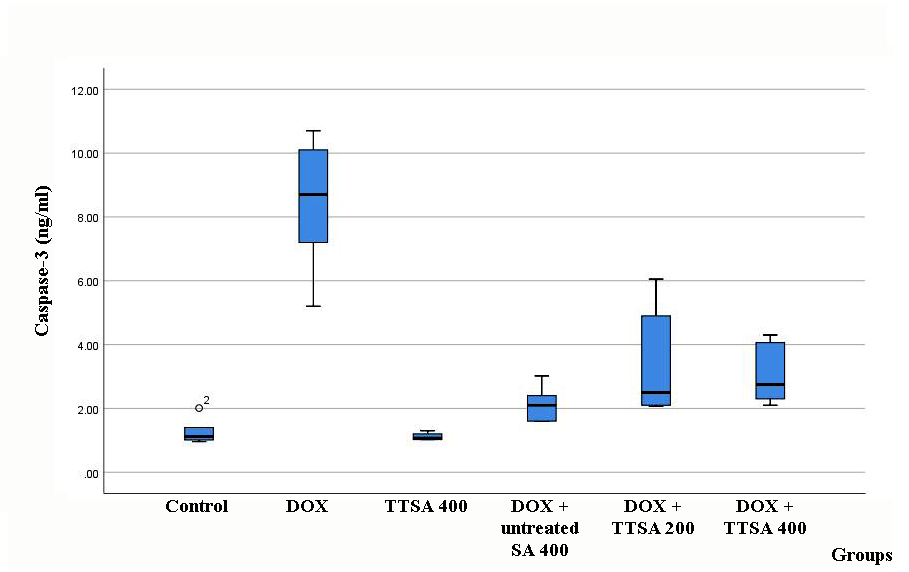

Supplement: Supplementary file 6 — High resolution image (TIF 83 kb) [file 11356_2023_26259_MOESM3_ESM.tif]

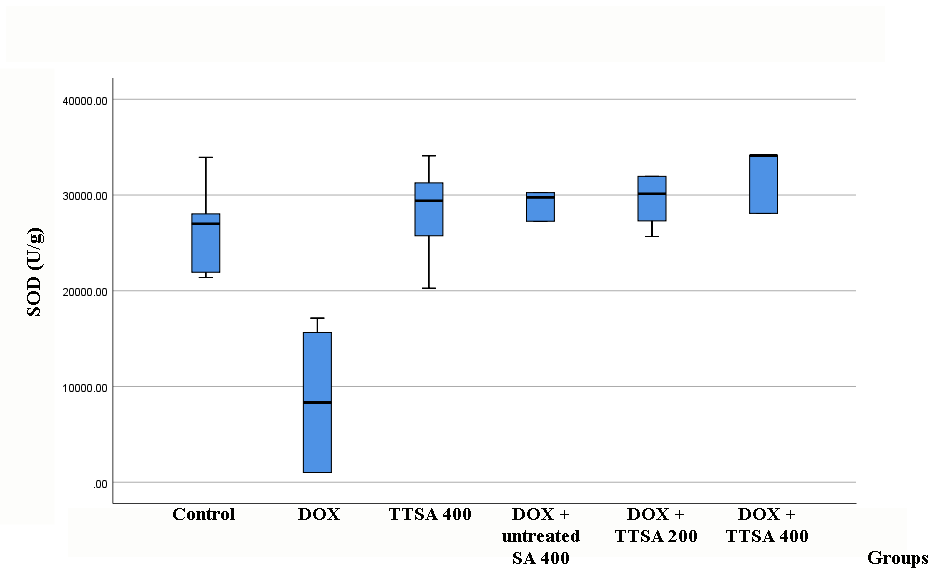

Supplement: Supplementary file 7 — Box–plot for concentrations of the enzyme superoxide dismutase (SOD) in all investigated animal groups.(PNG 9 kb) [file 11356_2023_26259_Fig13_ESM.png]

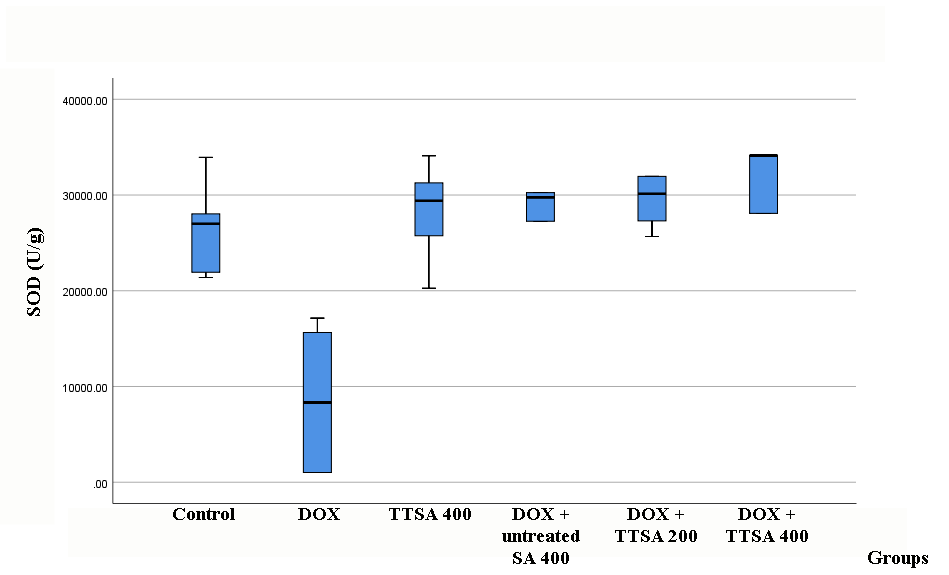

Supplement: Supplementary file 8 — High resolution image (TIF 66 kb) [file 11356_2023_26259_MOESM4_ESM.tif]

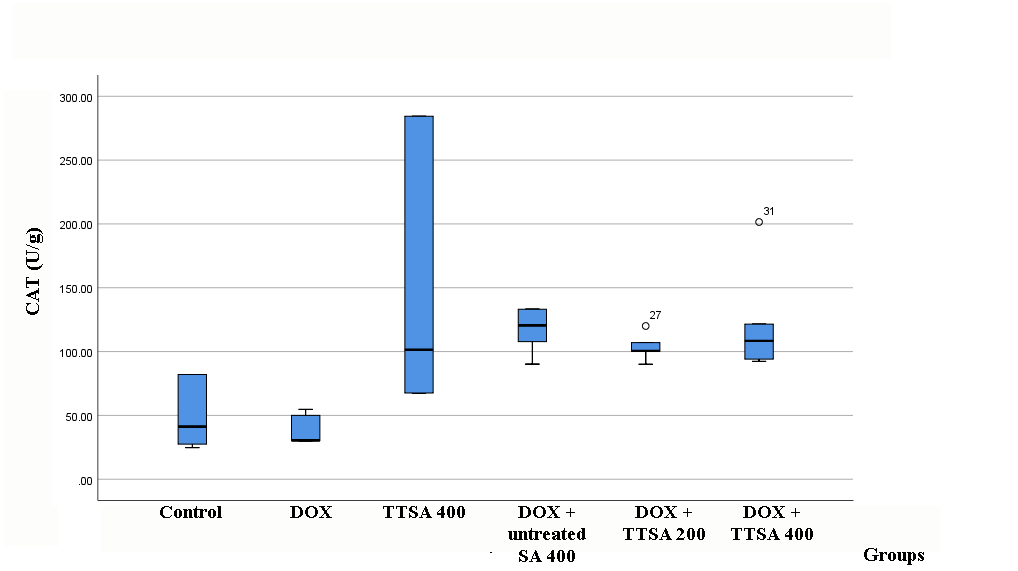

Supplement: Supplementary file 9 — Box–plot for concentrations of the enzyme catalase (CAT) in all investigated animal groups.(PNG 11 kb) [file 11356_2023_26259_Fig14_ESM.png]

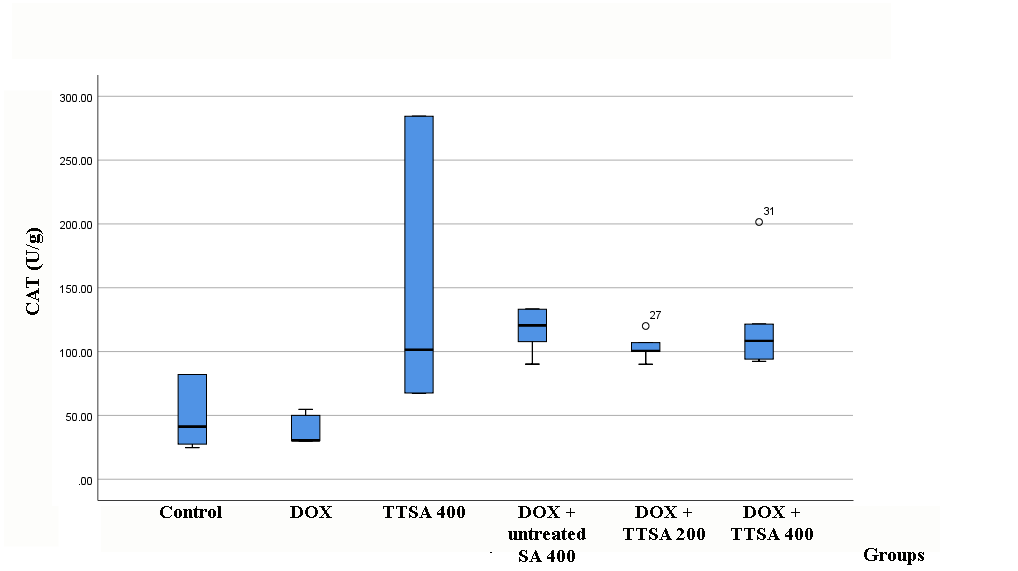

Supplement: Supplementary file 10 — High resolution image (TIF 69 kb) [file 11356_2023_26259_MOESM5_ESM.tif]
