# Supplementary material for: Seaweed Sargassum aquifolium extract ameliorates cardiotoxicity induced by doxorubicin in rats
Source: Environ Sci Pollut Res Int. 2023 Mar 28;30(20):58226–42. doi: 10.1007/s11356-023-26259-z (PMC10163098; doi:10.1007/s11356-023-26259-z)
Supplement: Supplementary file 11 — (DOCX 520 kb) [file 11356_2023_26259_MOESM6_ESM.docx]

**Fig. S6** Western blot analysis of MAPK-1 in all investigated animal groups using ImageJ.

**Area 14950**


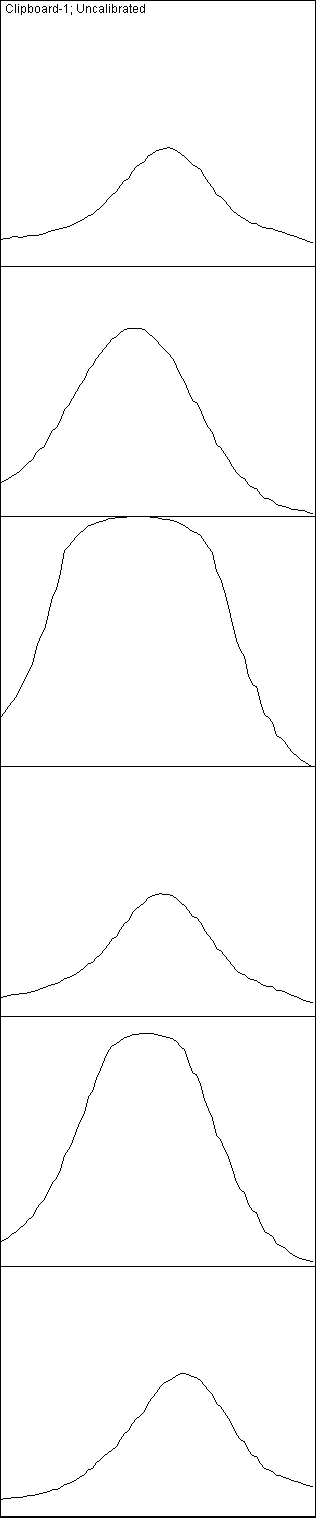


**Area 15604**

**TTSA 400**

**Area 104650**

**DOX + TTSA 400**

**Area 21674**

**Area 62790**

**DOX + untreated SA 400**

**Area 46344**

**Control**

**DOX**

**DOX + TTSA 200**
