# Supplementary material for: Seaweed Sargassum aquifolium extract ameliorates cardiotoxicity induced by doxorubicin in rats
Source: Environ Sci Pollut Res Int. 2023 Mar 28;30(20):58226–42. doi: 10.1007/s11356-023-26259-z (PMC10163098; doi:10.1007/s11356-023-26259-z)
Supplement: Supplementary file 12 — (DOCX 438 kb) [file 11356_2023_26259_MOESM7_ESM.docx]

**Fig. S7** Western blot analysis of p53 in all investigated animal groups using ImageJ.


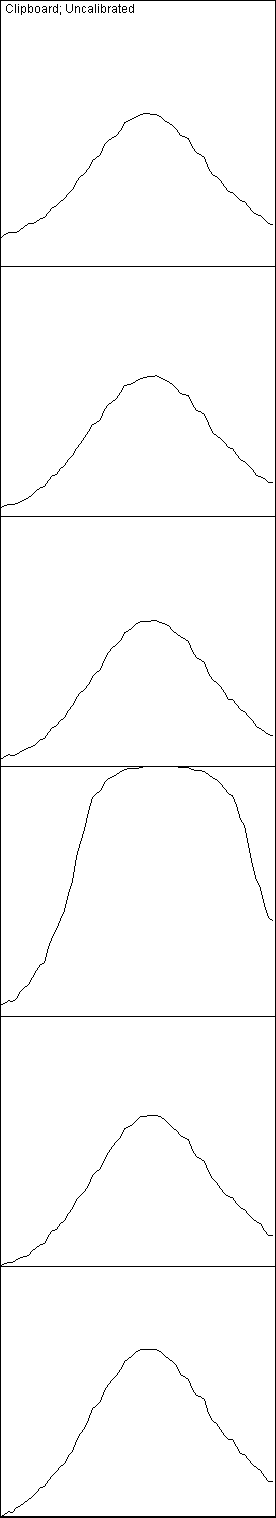


**Area 16438**

**Area 16604**

**Area 48760**

**Area 16134**

**Area 15988**

**Area 16533**

**TTSA 400**

**DOX + untreated SA 400**

**DOX**

**DOX + TTSA 400**

**DOX + TTSA 200**

**Control**
